# The Vermiform Appendix and Its Diseases

**Published:** 1905-04

**Authors:** 


THE VERMIFORM APPENDIX AND ITS DISEASES. By Howard A. Kelly, A. B., M. D., Professor of Gynecology in the Johns Hopkins University, Baltimore, and E. Hurdon, M. D., Assistant in Gynecology in the Johns Hopkins University, Baltimore. With 399 original illustrations, some in colors, and 3 lithographic plates. W. B. Saunders & Co., Philadelphia and London. Cloth, 820 pages, quarto, price, $10; sheep, $11.

This is one of the most important and valuable books that has appeared. It is a masterpiece of the bookmaker's art, and a great credit to the publishers. The great reputation of the author adds to the value of the book as a recognized authority. It is a history, really, of appendicitis and its treatment, and is literally an encyclopedia of information on the subject. It is not only a record of Dr. Kelly's extensive experience and observation, but a summary of that of other distinguished abdominal surgeons. The drawings—some of them micro-graphs—are, indeed, excellent, and show a high degree of art. The book is simply invaluable to every surgeon who ever expects to have to do with, not only the appendix, but with intestinal surgery. I recommend it without qualification. It is an epoch-marking book. D.

